# Rickettsial infection in *Amblyomma cajennense* ticks and capybaras (*Hydrochoerus hydrochaeris*) in a Brazilian spotted fever-endemic area

**DOI:** 10.1186/1756-3305-7-7

**Published:** 2014-01-05

**Authors:** Felipe S Krawczak, Fernanda A Nieri-Bastos, Fernanda P Nunes, João F Soares, Jonas Moraes-Filho, Marcelo B Labruna

**Affiliations:** 1 Department of Preventive Veterinary medicine and animal Health, Faculty of Veterinary Medicine, University of São Paulo, São Paulo, SP 05508-270, Brazil; 2 Veterinary and Technical Responsible by the Association Fazenda Vila Real de Itu - Rodovia Marechal Rondon Km 113.5, Itu, SP 13312-901, Brazil

**Keywords:** *Amblyomma cajennense*, *Rickettsia rickettsii*, Brazilian spotted fever, Capybara

## Abstract

**Background:**

Brazilian spotted fever (BSF), caused by the bacterium *Rickettsia rickettsii,* is the deadliest spotted fever of the world. In most of the BSF-endemic areas, capybaras (*Hydrochoerus hydrochaeris*) are the principal host for the tick *Amblyomma cajennense,* which is the main vector of BSF.

**Methods:**

In 2012, a BSF case was confirmed in a child that was bitten by ticks in a residential park area inhabited by *A. cajennense-*infested capybaras in Itú municipality, southeastern Brazil. Host questing *A. cajennense* adult ticks were collected in the residential park and brought alive to the laboratory, where they were macerated and intraperitoneally inoculated into guinea pigs. A tick-inoculated guinea pig that presented high fever was euthanized and its internal organs were macerated and inoculated into additional guinea pigs (guinea pig passage). Tissue samples from guinea pig passages were also used to inoculate Vero cells through the shell vial technique. Infected cells were used for molecular characterization of the rickettsial isolate through PCR and DNA sequencing of fragments of three rickettsial genes (*gltA*, *ompA*, and *ompB*). Blood serum samples were collected from 172 capybaras that inhabited the residential park. Sera were tested through the immunofluorescence assay using *R. rickettsii* antigen.

**Results:**

A tick-inoculated guinea pig presented high fever accompanied by scrotal reactions (edema and marked redness). These signs were reproduced by consecutive guinea pig passages. *Rickettsia* was successfully isolated in Vero cells that were inoculated with brain homogenate derived from a 3^rd^ passage-febrile guinea pig. Molecular characterization of this rickettsial isolate (designated as strain ITU) yielded DNA sequences that were all 100% identical to corresponding sequences of *R. rickettsii* in Genbank. A total of 83 (48.3%) out of 172 capybaras were seroreactive to *R. rickettsii,* with endpoint titers ranging from 64 to 8192.

**Conclusions:**

A viable isolate of *R. rickettsii* was obtained from the tick *A. cajennense,* comprising the first viable *R. rickettsi* isolate from this tick species during the last 60 years. Nearly half of the capybara population of the residential park was seroreactive to *R. rickettsii,* corroborating the findings that the local *A. cajennense* population was infected by *R. rickettsii.*

## Background

Brazilian spotted fever (BSF) is the deadliest spotted fever of the world. It has been reported in southeastern Brazil, where 591 laboratory-confirmed cases were reported from 1989 to 2008, resulting in 186 deaths (31.5% fatality rate) [1]. In the state of São Paulo, the most populated of Brazil, a total of 555 cases were laboratory confirmed from 1985 to 2012, with 225 deaths (40.5% fatality rate) (official data from the São Paulo State Health Office, available at http://www.cve.saude.sp.gov.br/htm/cve_fmb.html). The disease is caused by the bacterium *Rickettsia rickettsii,* which also causes severe cases of spotted fever in other American countries, including the United States, where the disease is called Rocky Mountain spotted fever (RMSF). In contrast to the high fatality rates of BSF in southeastern Brazil, RMSF has been reported at 5-10% fatality rates in the United States [2]. The reasons for such a contrast are still speculative; however, two studies have showed genetic differences between *R. rickettsii* strains from South and North Americas [3,4], highlighting the possibility that more virulent strains circulate in South America, as recently suggested by different authors [4-6].

In Brazil, *R. rickettsii* is considered to be transmitted to humans by two tick species: *Amblyomma aureolatum* in the metropolitan area of São Paulo [7], and by *Amblyomma cajennense* in the remaining BSF-endemic areas of southeastern Brazil [8,9]. In the metropolitan area of São Paulo, 1-10% infection rates by *R. rickettsii* have been reported in *A. aureolatum* tick populations [7,10]. In these areas, *R. rickettsii* is considered to be successfully maintained in tick populations through very efficient transovarial and transstadial transmissions [11]. However, because *R. rickettsii* elicits some deleterious effect to *A. aureolatum* engorged females, horizontal transmission through the participation of amplifier vertebrate hosts for the formation of new lineages of infected ticks seems to be crucial in a long term scenario for maintenance of *R. rickettsii* in *A. aureolatum* populations [11]. Competent amplifier hosts are still unknown in the *A. aureolatum* transmission areas [7].

Similarly to that previously reported for *A. aureolatum,* the infection by *R. rickettsii* also elicits deleterious effects on *A. cajennense* engorged females [12]*.* However, *R. rickettsii* is not very efficiently maintained in *A. cajennense* populations through transovarial and transstadial transmissions, since less than 50% of the infected females pass the agent vertically, and when they do, less than 50% of the offspring become infected [12]. For this reason, the participation of vertebrate amplifier hosts to create new lineages of infected ticks, in order to avoid the disappearance of *R. rickettsii* from the tick population, is likely to be frequently needed in the *A. cajennense-*BSF transmission areas [12]*.* In this case, capybaras (*Hydrochoerus hydrochaeris*) have played a crucial role in BSF-endemic areas of southeastern Brazil [8,12,13]. Besides being the most important host for all parasitic stages of *A. cajennense* in these endemic areas, capybaras are efficient amplifier hosts of *R. rickettsii* for *A. cajennense* ticks, as demonstrated in a recent study [14].

During the first half of the 20^th^ century, *A. cajennense* was first incriminated as a vector of *R. rickettsii* due to its epidemiological association with the BSF human cases, and because a number of *R. rickettsii* isolates were obtained from this tick species, through the inoculation of tick homogenates into guinea pigs [15-18]. Since then, no additional viable isolates of *R. rickettsii* have been obtained from *A. cajennense.* While a few recent studies detected *R. rickettsii* DNA in *A. cajennense* ticks [9,19], several other ones failed to detect a single *R. rickettsii-*infected tick by molecular methods among hundreds to thousands of *A. cajennense* ticks collected from BSF-endemic areas [20-23]. These findings corroborate the above mentioned experimental studies, which showed that *A. cajennense* is not very efficient to sustain a *R. rickettsii* infection, and for this reason, finding a *R. rickettsii-*infected *A. cajennense* tick has been a very difficult task. Even under such circumstances, *A. cajennense* still assumes a major role as vector of *R. rickettsii* to humans just because this tick is the most common human-biting tick in southeastern Brazil, where humans are frequently infested by dozens to hundreds of individuals in each infestation episode [20,24,25].

In the present study, we investigated the circulation of *R. rickettsii* among capybaras and ticks in one area where a laboratory-confirmed case of BSF had just occurred. During the study, we obtained a viable isolate of *R. rickettsii* from *A. cajennense* ticks, comprising the first isolate established from this tick species during the last 60 years.

## Methods

### Study area and tick collection

This study was performed in a residential park area at Itú Municipality, state of São Paulo, southeastern Brazil. The residential park had an area of 484 ha, which contained four lakes, and ≈ 400 homes interposed by conserved forest areas. In September 2012, a severe case of BSF occurred in a child few days after being infested by ticks while playing near one of the lakes of the residential park, inhabited by free-ranging capybaras. After two weeks under hospitalization and therapy with chloramphenicol, the child had the infection resolved. In October 2012, we visited the residential park for collection of free-living ticks by using 20 CO_2_ traps armed at capybara common places (including the site where the child got tick-infested) as previously described [26]. Thousands of *Amblyomma* unfed nymphs, and hundreds of *Amblyomma* unfed adult ticks were captured by the CO_2_ traps. Only adult ticks were taken alive to the laboratory, where they were incubated at 35°C and 95-100% relative humidity for 48 hours, prior to being processed for rickettsial isolation.

### Isolation of rickettsiae

After incubation for 48 h in the laboratory, live adult ticks were counted, identified to species, and separated into 660 *A. cajennense* and 42 *Amblyomma dubitatum* ticks*.* The 660 *A. cajennense* ticks were used to form 7 pools; 6 pools contained 100 ticks, 1 pool contained 60 ticks. The 42 *A. dubitatum* ticks formed a single pool. Ticks of each pool, still alive, were disinfected for 10 min in iodine alcohol, followed by several washes in sterile water, and then macerated all together into brain-heart infusion broth (BHI) in a sterile mortar with the aid of a sterile alundum. The resultant tick homogenate (≈7 mL) was inoculated intraperitoneally into an adult male guinea pig. Since we had 8 tick pools, we used 8 guinea pigs, one per tick pool. Guinea pigs had their rectal temperature measured daily until the 21st day post inoculation (DPI), when they were bled through cardiac puncture, and their sera were individually tested for the presence of anti-*R. rickettsii* antibodies through the immunofluorescence assay (IFA) using *R. rickettsii* antigen, as previously described [12]. If any of the tick-inoculated guinea pigs presented high fever (rectal temperature >40.0°C) for 3 consecutive days within the 21-day period, it was euthanized at the 3^rd^ febrile day and its internal organs (liver, spleen, lungs, brain) were macerated into BHI and the resultant homogenate was inoculated into additional guinea pigs (guinea pig passage). This procedure was repeated with additional guinea pigs; in each guinea pig passage, samples of the internal organs were frozen directly at -80°C, and then in liquid nitrogen, in order to cryopreserve the isolate before it could be adapted to *in vitro* tissue culture.

BHI-brain homogenate obtained from a 3rd passage-febrile guinea pig was divided into four 200 μl aliquots; each one was inoculated into one shell vial containing a monolayer of confluent Vero cells, as previously described [27]. After inoculation, the shell vials were centrifuged for 1 h at 700 *g* and 22°C. Then the monolayer was washed once with Roswell Park Memorial Institute medium (RPMI; Gibco, Carlsbad, CA) containing 10% bovine calf serum (Hyclone, Logan, UT) and subsequently incubated at 28 or 34°C (two shell vials at each temperature). Every 3 days, the medium was switched to new medium, and the aspirated medium was checked by Giménez staining for the presence of *Rickettsia*-like organisms. If the result was positive, the monolayer of the shell vial was harvested and inoculated into a 25-cm^2^ flask containing a monolayer of confluent uninfected Vero cells. Cells in the 25-cm^2^ flask were checked by Giménez staining until >90% of them were infected, when they were harvested and inoculated into 75-cm^2^ flasks of Vero cells. The level of infection of cells was monitored by Giménez staining of scraped cells from the inoculated monolayer. The rickettsial isolate was considered to be established in the laboratory after at least three passages through 75-cm^2^ Vero cell flasks, each achieving a proportion >90% of infected cells [28].

For molecular characterization of the rickettsial isolate, DNA from the third infected cell passage was extracted by using the DNAeasy Blood and Tissue Kit (Qiagen, Valencia, California), and tested by a battery of different PCR protocols, with two primer pairs (CS-78 and CS-323; CS-239 and CS-1069) targeting two overlapping fragments of the rickettsial *gltA* gene [28], primers Rr190.70 F and Rr190.701R targeting a portion of the rickettsial *ompA* gene [29], and primers 120.M59 and 120-807 targeting a portion of the rickettsial *ompB* gene [30]. All PCR products were DNA sequenced, and the resultant sequences were compared with GenBank data by BLAST analysis.

### Capybaras

Because the *A. cajennense* population of the residential park was sustained primarily by capybaras (no other important host for this tick species, such as horses, tapirs or pigs, was present in the area), the residential park was legally authorized to cull its entire capybara population with the aim to reduce the *A. cajennense* population to minimal levels within the residential park area. For this purpose, from December 2012 to May 2013, a total of 172 capybaras were collected by corrals according to Moreira *et al*. [31], and before been culled, individual blood samples were collected and sent to the laboratory for serological analysis. Capybara sera were individually tested through the IFA using *R. rickettsii* antigen, as previously described [14]. Briefly, capybara sera were diluted in 2-fold increments with phosphate-buffered saline (PBS), starting from the 1:64 dilution. A fluorescein isothiocyanate-labeled sheep anti-capybara IgG (CCZ, São Paulo/SP, Brazil) was used as conjugate. For each sample, the endpoint IgG titer reacting with *R. rickettsii* strain Taiaçu crude antigen was determined. In each slide, a serum previously shown to be non-reactive (negative control) and a known reactive serum (positive control), both from the study of Souza *et al*. [14], were tested at the 1:64 dilution. The proportions of seroreactive capybaras were compared between three age categories (infant, juvenile, or adult) and gender (male or female) by the Chi-square test. Values were considered significant at *P* < 0.05.

### Ethical approval

This work was authorized by the Environment State Secretary of the state of São Paulo (authorization no. 96/2012) and was approved by the Ethical Committee of Animal Use of the Faculty of Veterinary Medicine of the University of São Paulo (protocol No. 3104/2013).

## Results

Among the 8 guinea pigs inoculated with adult tick homogenate, 2 animals (one inoculated with 100 *A. cajennense,* and one inoculated with 60 *A. cajennense*) died in less than 24 hours, and were discarded from the study. Among the remaining 6 guinea pigs (each of 5 inoculated with 100 *A. cajennense* ticks; 1 inoculated with 42 *A. dubitatum*), only one (inoculated with *A. cajennense*) presented high fever at the 3^rd^ DPI, which persisted until the 5^th^ DPI, when the animal was euthanized and its internal organs were used to inoculate 3 additional guinea pigs, which all developed high fever starting at 3-5 DPI. These animals were euthanized at the 3^rd^ febrile day and their internal organs were used to inoculate 2 additional guinea pigs. A total of 4 guinea pig passages was performed, totaling 9 inoculated guinea pigs, which all developed high fever (>40.0°C) that started 3 to 6 DPI. In all cases, fever was accompanied by scrotal reactions (edema and marked redness); a single guinea pig from the 3rd passage was not euthanized; in this case, the animal presented high fever from the 5^th^ to the 9^th^ DPI. When this animal was tested by serology at the 21th DPI, it presented a 8,192 endpoint titer to *R. rickettsii.* The remaining guinea pigs that were primarily inoculated with tick homogenate (each of 4 inoculated with 100 * A. cajennense*, 1 inoculated with 42 *A. dubitatum*) remained afebrile (rectal temperature <39.5°C) until 21 DPI, when they were bled and were shown to be seronegative to *R. rickettsii.*

Rickettsiae were successfully isolated in Vero cells that were inoculated with brain homogenate derived from a 3^rd^ guinea pig passage of the *A. cajennense* rickettsial isolate. A total of four Vero cell passages has been done, always resulting in 100% infected cells 5 to 8 days after inoculation of the monolayer. The rickettsial isolate was designated as strain ITU. DNA of strain ITU-infected cells at third passage was subjected to a battery of PCR protocols, which successfully amplified fragments of the rickettsial genes *gltA*, *ompA*, and *ompB*. We sequenced 1068, 590, and 763 nucleotides of the *gltA*, *ompA*, and *ompB* genes, respectively, which all showed 100% identity to corresponding sequences in the *R. rickettsii* genomes from Brazil [GenBank: CP003305] and Colombia [GenBank: CP003306]. The GenBank nucleotide sequence accession numbers of the partial sequences of *R. rickettsii* strain ITU generated in this study are KF742602 for the *gltA* gene, KF742603 for the *ompA* gene, and KF742604 for the *ompB* gene.

**Figure 1:**
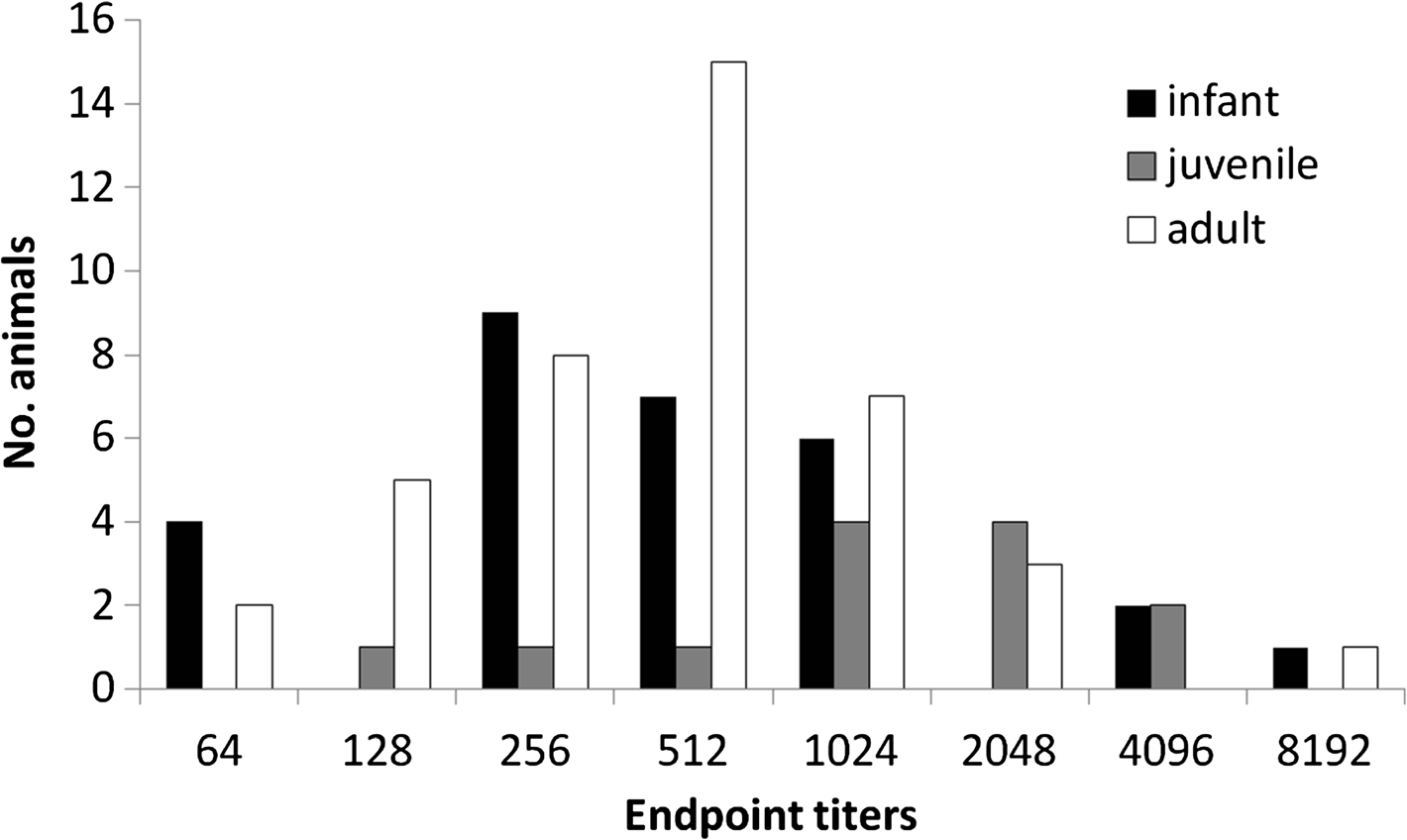
**Anti-*Rickettsia rickettsii *** **endpoint titers of IgG antibodies in capybaras of three age groups (infant, juvenile, and adult) from a residential park at Itu Municipality, state of São Paulo, Brazil.**

A total of 172 capybaras were sampled in the residential park. From these, 83 (48.3%) were seroreactive to *R. rickettsii* antigens. The proportions of seroreactive animals were statistically similar among different age groups (Table 1). Similarly, the proportions of seroreactive animals were statistically similar between male (46/85; 54.1%) and female (37/87; 42.5%) capybaras (chi-square: 1.87; d.f. 1; *P =* 0.17). Seroreactive capybaras presented endpoint titers to *R. rickettsii* ranging from 64 to 8192. The most frequent endpoint titer was 256 for infant animals, 1024 and 2048 for juveniles, and 512 for adult capybaras (Figure 1).

**Table 1:** **Number of capybaras seroreactive to *Rickettsia rickettsii *** **according to age category**

**Age category**	**No. tested**	**No. seroreactive (%)**
Infant	62	29 (46.8)a
Juvenile	35	13 (37.1)a
Adult	75	41 (54.7)a
Total	172	83 (48.3)

a: the proportions of seroreactive animals were not significantly different between age categories (chi-square: 3.02; d.f. 2; *P =* 0.22).

## Discussion

A viable isolate of *R. rickettsii* was obtained from the tick *A. cajennense* collected from a BSF-endemic area. To achieve such results, we initially performed intraperitoneal inoculation of tick homogenate into guinea pigs, a method that was widely used during the first half of the 20^th^ century, when all previous isolates of *R. rickettsii* from *A. cajennense* were achieved [15-18,32-34]. With such procedure, we were able to sample hundreds of ticks with relatively few efforts, if compared to more contemporary methods that usually test ticks individually or in pools of few specimens by inoculating cell culture, such as the shell vial technique [28,35,36]. Indeed, the increasing use of the shell vial technique has allowed isolation of various *Rickettsia* species that could not be isolated by inoculation of guinea pigs with field-collected samples, just because most of the *Rickettsia* species are not pathogenic for this animal species [37]. On the other hand, because guinea pig inoculation remains an efficient and simple method for isolation of *R. rickettsii* from field-collected ticks*,* it should be adopted in more studies in Latin America, where *R. rickettsii* remains as the agent of a neglected, deadly bacterial disease. In fact, at least two recent studies have used guinea pig inoculation of patient samples for confirmatory diagnostic of RMSF fatal cases in Colombia and Costa Rica [38,39].

Only one guinea pig, inoculated with 100 *A. cajennense* adult ticks, developed rickettsiosis that was shown to be caused by *R. rickettsii.* This result indicates that at least one *R. rickettsii-*infected tick was present in the tick pool that was used to inoculate this guinea pig. Because other 4 guinea pigs, each inoculated with 100 *A. cajennense,* remained afebrile and seronegative during the study, we can infer a minimal infection rate of 0.2% (1/500) for the *A. cajennense* population tested in the present study. This low rate is quite expected since several recent studies have failed to detect *R. rickettsii*-infected ticks from BSF-endemic areas [20-23], whereas two other studies performed in one endemic area detected *R. rickettsii* DNA in at least 1 (1.28%) out of 78 *A. cajennense* ticks collected during 2003-2004, and 2 (0.5%) out of 400 *A. cajennense* ticks collected during 2006-2008 [9,19]. Since human infestation by *A. cajennense* is very common in southeastern Brazil [20,25], very low *R. rickettsii-*infection rates among *A. cajennense* populations from BSF-endemic areas should be a major reason for the low incidence of the disease (usually 0.1 case per 100,000 persons) in these areas [40].

For the present study, we collected unfed ticks from the environment and incubated them at 35°C for 48 h prior to guinea pig inoculation. This procedure was adopted because previous studies reported that injection of triturated, unfed, *R. rickettsii-*infected ticks into guinea pigs did not lead to disease, but caused seroconversion. However, feeding the ticks for a short time or keeping them at an elevated temperature (24-48 h at 35-37°C before trituration and inoculation into non-immune guinea pigs) resulted in clinically manifest disease [41-43]. Finally, this incubation at higher temperature for 48 h might also have induced rickettsial multiplication within ticks [44], and therefore, enhanced the chances of rickettsial isolation, especially because we worked with pools of up to 100 unfed ticks.

The 172 capybaras tested by serology represented at least 90% of the whole capybara population of the residential park. Nearly half of this capybara population was seroreactive to *R. rickettsii,* similarly to what has been found in other BSF-endemic areas, where 40-100% of horses or capybaras (main hosts of *A. cajennense*) have been found to be seroreactive to *R. rickettsii*[20,45,46]*.* In a recent study [14], capybaras experimentally infected with *R. rickettsii* via infected *A. cajennense* ticks developed high antibody endpoint titers (8,192-32,768) from 30 to 146 days post infestation. Because at least two capybaras of the present study also showed high antibody endpoint titers (8192) (Figure 1), we can infer that they were exposed to *R. rickettsii-*infected ticks during few months prior to being sampled. On the other hand, capybaras with lower antibody titers could be in a phase of ascending (recent infection) or descending (earlier infection) antibody titers to *R. rickettsii.* Finally, the overall lower titers observed in infant capybaras of the present study could be related to passive immunity, whereas overall higher endpoint titers in juvenile and adult capybaras could be related to a past active immunity elicited by transmission of *R. rickettsii* by ticks.

The fact that nearly half of the capybara population of the residential park was seronegative to *R. rickettsii* means that new lineages of infected ticks could be created in a short term, as susceptible animals would develop rickettsemia after a primary infection via infected ticks. Therefore, the procedure of the residential park to cull its entire capybara population was reasonable, in order to prevent new human cases of BSF in the area, where no other host species (i.e., horses, tapirs, pigs) that could sustain a *A. cajennense* population was present.

## Conclusions

A viable isolate of *R. rickettsii* was obtained from the tick *A. cajennense,* comprising the first viable *R. rickettsi* isolate from this tick species during the last 60 years. Nearly half of the capybara population of the residential park was seroreactive to *R. rickettsii,* corroborating the findings that the local *A. cajennense* population was infected by *R. rickettsii.*

## Competing interests

The authors declare that they have no competing interests.

## Authors’ contributions

FSK performed tick field study, lab experiments, processed the data, and drafted the manuscript. FAN-B and JFS conducted lab experiments, processed the data, and revised the manuscript. FPN performed capybara field study and revised the manuscript. MBL contributed to study design, field study, data analysis and interpretation, and revised the manuscript. All authors read and approved the final manuscript.
